# Average Hazard as Harmonic Mean

**DOI:** 10.1002/pst.70009

**Published:** 2025-03-10

**Authors:** Yasutaka Chiba

**Affiliations:** ^1^ Clinical Research Center Kindai University Hospital Osaka Japan

**Keywords:** arithmetic mean, average hazard, average of hazards, harmonic mean, person‐time incidence rate, survival analysis

## Abstract

A new measure was recently developed in the context of survival analysis that can be interpreted as a weighted arithmetic mean of the hazards with the survival function as the weight. However, when the average hazard is desired, it is more appropriate to use the harmonic mean rather than the arithmetic mean. Therefore, in this article, we derive the average hazard as a harmonic mean version of the expectation for hazards and show it to be equal to the previous weighted arithmetic mean. Furthermore, we demonstrate that the average hazard should be estimated using only the times at which the event is observed, while previous studies have allowed estimating the average hazard even when the truncation time is set to a time at which the event is not observed.

## 1 Introduction

In clinical studies with a time‐to‐event outcome as the endpoint, the hazard ratio derived by the Cox regression model is commonly applied. However, the Cox regression model is based on a proportional hazard assumption, and it is generally difficult to provide an appropriate interpretation of the calculated hazard ratio [1]. Moreover, most clinical studies to date have continued to ignore the appropriate interpretation.

To solve this problem, Snapinn et al. [2] recently proposed a new measure called the generalized hazard, which is derived as the expected number of events within the total survival time. Jackson et al. [3] demonstrated that this measure can be interpreted as a weighted arithmetic mean of hazards with the survival function as the weight, which was proposed by Uno and Horiguchi [4]. Since the generalized hazard can be estimated nonparametrically without any assumptions, it is expected that this measure will be applied to future clinical studies. At present, the generalized hazard has not been recognized sufficiently, and further discussion may be required.

We begin with the previously used interpretation of the generalized hazard as the weighted arithmetic mean. However, as is well known, it is more appropriate to derive the average rate using the harmonic mean rather than the arithmetic mean. As a commonplace example, suppose that an airplane flies from city A to city B at a constant rate of $x_{1}$ (km/h) and on the return trip, the same airplane flies at a constant rate of $x_{2}$ (km/h) [5]. In this example, it is more appropriate to derive the average rate by the harmonic mean, $2/\left(1/x_{1}+1/x_{2}\right)$, rather than by the arithmetic mean, $\left(x_{1}+x_{2}\right)/2$. Although the harmonic mean can be expressed by the weighted arithmetic mean, it is somewhat unnatural to consider the weighted arithmetic mean to derive the average rate. According to Lindstrom [5], the arithmetic mean no longer represents “the average rate,” but rather “the average of the rates.” Following this, we represent “the average hazard” as the harmonic mean of the hazards and “the average of the hazards” as the arithmetic mean of the hazards.

In this article, we derive the average hazard as the harmonic mean version of the expectation for hazards and show it to be equal to the generalized hazard. In addition, we suggest that the average hazard should be estimated using only the time at which the event is observed. To justify this suggestion, we demonstrate that a contradiction occurs when the truncation time is set to a time at which the event is not observed.

## 2 Average Hazard

As a preliminary step, we begin by showing that the traditional person‐time incidence rate can be regarded as a harmonic mean. Consider a situation in which all n subjects experience the event, with $d_{i}$ subjects experiencing it at time $t_{i}$ (i=1,…,k). In this scenario, the person‐time incidence rate is formulated as

$$\sum_{i=1}^{k}d_{i}/\sum_{i=1}^{k}d_{i}t_{i}$$

where $\sum_{i=1}^{k}d_{i}=n$ [6]. It can easily be shown that this is equivalent to

(1) $$\sum_{i=1}^{k}d_{i}/\sum_{i=1}^{k}\frac{d_{i}}{\hat{h}\left(t_{i}\right)}$$

where $\hat{h}\left(t_{i}\right)=d_{i}/\left\{r_{i}\left(t_{i}-t_{i-1}\right)\right\}$ with $r_{i}=\sum_{j=i}^{k}d_{j}$, which is the number of subjects in the risk set at time $t_{i}$, and $t_{0}=0$. Since $\hat{h}\left(t_{i}\right)$ represents the slope between $t_{i-1}$ and $t_{i}$ for the Nelson–Aalen plot of the cumulative hazard function, it serves as a crude estimator of the hazard [7]. Thus, in the absence of censoring, the person‐time incidence rate can be interpreted as a weighted harmonic mean of $\hat{h}\left(t_{i}\right)$, which corresponds to the harmonic mean version of the expectation. We note that $\hat{h}\left(t_{i}\right)$ cannot be defined at a time at which the event is not observed [8]. We also note that the harmonic mean (1) can be expressed by a weighted arithmetic mean:

$$\frac{\sum_{i=1}^{k}r_{i}\left(t_{i}-t_{i-1}\right)\hat{h}\left(t_{i}\right)}{\sum_{i=1}^{k}r_{i}\left(t_{i}-t_{i-1}\right)}$$

In the remainder of this article, we address a scenario involving censoring. In this scenario, $\sum_{i=1}^{k}d_{i}$ subjects of n experience the event, while $n-\sum_{i=1}^{k}d_{i}$ are censored. Here, $r_{i}$ is replaced by $\sum_{j=i}^{k}d_{j}$ plus the number of subjects who were censored for $t\geq t_{i}$.

As a natural extension of the harmonic mean (1), we present the average hazard up to time τ for n→∞. We consider the average hazard as the harmonic mean version of the expectation for hazards over the interval 0≤t≤τ. Under this consideration, the average hazard is formulated as

(2) $$\mathrm{AH}\left(\tau \right)=\int_{0}^{\tau }f\left(t\right)\mathrm{dt}/\int_{0}^{\tau }\frac{f\left(t\right)}{h\left(t\right)}\mathrm{dt}$$

where $h\left(t\right)$ and $f\left(t\right)$ denote the hazard function and probability density function, respectively. Using the relationship $f\left(t\right)=h\left(t\right)S\left(t\right)$, where $S\left(t\right)$ is the survival function, $\mathrm{AH}\left(\tau \right)$ can be expressed as

$$\mathrm{AH}\left(\tau \right)=\frac{\int_{0}^{\tau }f\left(t\right)\mathrm{dt}}{\int_{0}^{\tau }S\left(t\right)\mathrm{dt}}=\frac{1-S\left(\tau \right)}{\int_{0}^{\tau }S\left(t\right)\mathrm{dt}}$$

This “average hazard” based on the harmonic mean is equivalent to the generalized hazard proposed by Snapinn et al. [2], which can be interpreted as the weighted “average of hazards” (i.e., arithmetic mean) of $h\left(t\right)$ with $S\left(t\right)$ as the weight [3]:

(3) $$\int_{0}^{\tau }S\left(t\right)h\left(t\right)\mathrm{dt}/\int_{0}^{\tau }S\left(t\right)\mathrm{dt}$$

## 3 Estimation of Average Hazard

For finite n, previous studies [2, 4] have proposed to estimate $\mathrm{AH}\left(\tau \right)$ using the Kaplan–Meier estimator $\hat{S}\left(t\right)$, as follows:

(4) $$\hat{\mathrm{AH}}\left(\tau \right)=\frac{1-\hat{S}\left(\tau \right)}{\sum_{j=1}^{i}\hat{S}\left(t_{j-1}\right)\left(t_{j}-t_{j-1}\right)+\hat{S}\left(t_{i}\right)\left(\tau -t_{i}\right)}$$

where $t_{i}=\max\left\{t_{j}\left|t_{j}\leq \tau \right.\right\}$ for j=1,…,k, and $\hat{S}\left(t_{j}\right)=\prod_{l=0}^{j}\left(1-d_{l}/r_{l}\right)$. Here, we show that Formula (4) is incorrect for $t_{i}<\tau <t_{i+1}$ and that the average hazard should be estimated using only the times at which the event is observed.

### 3.1 Numerical Example

**FIGURE 1:**
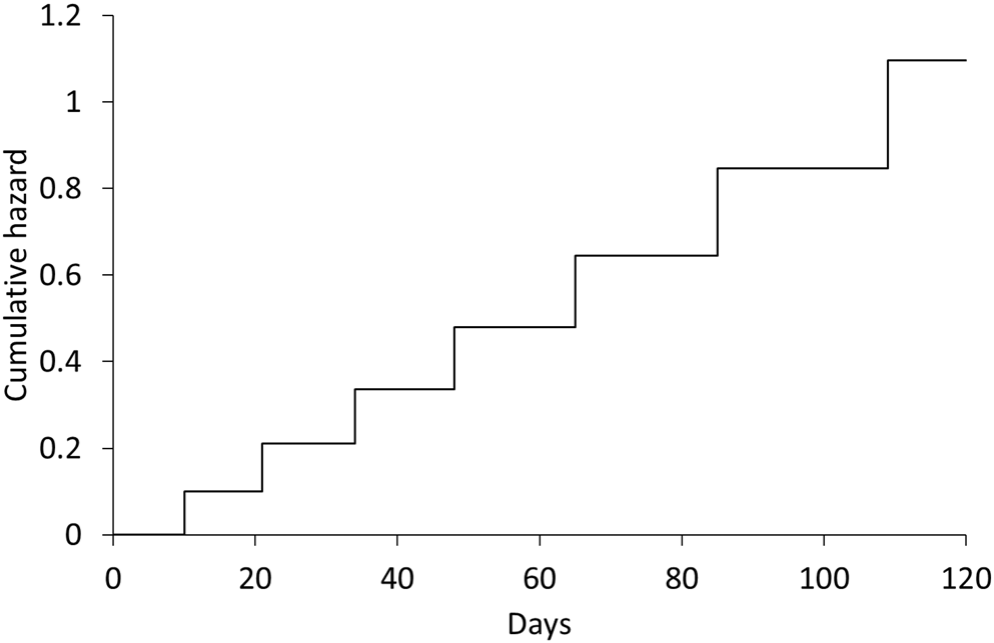
Nelson–Aalen plot of cumulative hazard function.

**FIGURE 2:**
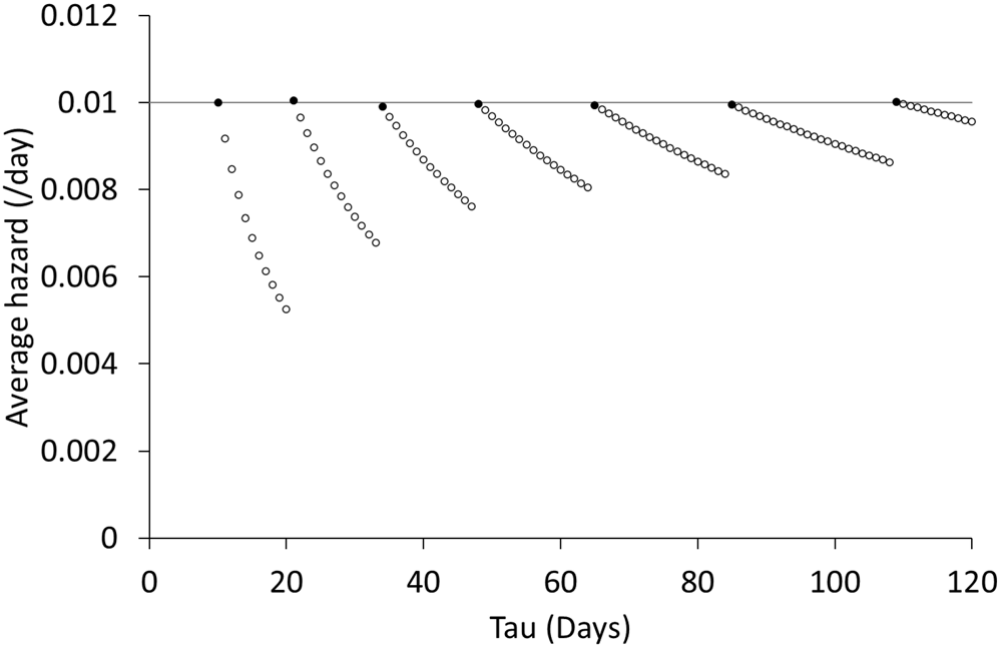
Average hazards from Formula (4) for τ=10,11,…,120, where the filled circles represent the τ value being the same as the time of the event occurrence and the open circles represent it being different.

First, we demonstrate that Formula (4) may not estimate the average hazard appropriately when $t_{i}<\tau <t_{i+1}$, through the following simple numerical example:

$$10,21,34,48,65,85,109,120^{*},120^{*},120^{*}\;\left(\text{days}\right)$$

which represent the numbers of days until the event occurrence or censoring, where the asterisk represents the censor. We can draw Figures 1 and 2 from this data set. Figure 1 shows the Nelson–Aalen plot of the cumulative hazard function, and Figure 2 summarizes $\hat{\mathrm{AH}}\left(\tau \right)$ if the truncation time is set to τ=10,11,…,120. In Figure 2, the filled circles represent $\hat{\mathrm{AH}}\left(\tau \right)$ for $\tau =t_{i}$ (i=1,…,7), where $t_{i}$ is the time of the event occurrence, and the open circles represent $\hat{\mathrm{AH}}\left(\tau \right)$ for $\tau \neq t_{i}$.

Figure 1 shows that the hazard $h\left(t\right)$ is approximately constant over time, because the slopes between $t_{i-1}$ and $t_{i}$ are approximately constant among i=1,…,7. When the hazard is constant over time, the average hazard should also be constant over time. Nevertheless, as shown in Figure 2, the values of $\hat{\mathrm{AH}}\left(\tau \right)$ are not constant for $\tau \neq t_{i}$, while those are approximately constant for $\tau =t_{i}$. This observation shows that the average hazard may not be estimated appropriately when the truncation time is set to a time at which the event is not observed.

As an example, we consider the case of τ=20. In this case, Formula (4) derives $\hat{\mathrm{AH}}\left(20\right)=1/190$, where $\hat{\mathrm{AH}}\left(10\right)=\hat{h}\left(10\right)=1/100$. Our question is: what is the value of $\hat{h}\left(20\right)$? Until a satisfactory answer is given, it cannot be justified that Formula (4) is correct.

### 3.2 Theoretical Aspects

Next, we show that Formula (4) is incorrect for $t_{i}<\tau <t_{i+1}$, using a proof by contradiction. For form's sake, Formula (4) can be expressed as a natural estimator of Formula (2):

$$\hat{\mathrm{AH}}\left(\tau \right)=\frac{\sum_{j=1}^{i}\hat{f}\left(t_{j}\right)\left(t_{j}-t_{j-1}\right)+\hat{f}\left(\tau \right)\left(\tau -t_{i}\right)}{\sum_{j=1}^{i}\frac{\hat{f}\left(t_{j}\right)\left(t_{j}-t_{j-1}\right)}{\hat{h}\left(t_{j}\right)}+\frac{\hat{f}\left(\tau \right)\left(\tau -t_{i}\right)}{\hat{h}\left(\tau \right)}}$$

where $\hat{f}\left(t_{j}\right)=\hat{h}\left(t_{j}\right)\hat{S}\left(t_{j-1}\right)$ and

$$\hat{h}\left(t_{j}\right)=\frac{\hat{S}\left(t_{j-1}\right)-\hat{S}\left(t_{j}\right)}{\hat{S}\left(t_{j-1}\right)\left(t_{j}-t_{j-1}\right)}=\frac{d_{j}}{r_{j}\left(t_{j}-t_{j-1}\right)}$$

Under the assumption that Formula (4) is correct for $t_{i}<\tau <t_{i+1}$, this formula for $\hat{\mathrm{AH}}\left(\tau \right)$ implies that $\hat{f}\left(\tau \right)=0$ and $\hat{f}\left(\tau \right)/\hat{h}\left(\tau \right)>0$ because $\hat{S}\left(\tau \right)=\hat{S}\left(t_{i}\right)$ and $\hat{S}\left(t_{i}\right)\left(\tau -t_{i}\right)>0$ in Formula (4). However, it is obvious that $\hat{f}\left(\tau \right)=0$ contradicts $\hat{f}\left(\tau \right)/\hat{h}\left(\tau \right)>0$. Therefore, the assumption is false; that is, Formula (4) is incorrect for $t_{i}<\tau <t_{i+1}$. Since this contradiction does not occur for $\tau =t_{i}$, the average hazard should be estimated using only the times at which the event is observed. In conclusion, Formula (4) should be replaced by

$$\hat{\mathrm{AH}}\left(t_{i}\right)=\frac{\sum_{j=1}^{i}\left\{\hat{S}\left(t_{j-1}\right)-\hat{S}\left(t_{j}\right)\right\}}{\sum_{j=1}^{i}\frac{\hat{S}\left(t_{j-1}\right)-\hat{S}\left(t_{j}\right)}{\hat{h}\left(t_{j}\right)}}=\frac{1-\hat{S}\left(t_{i}\right)}{\sum_{j=1}^{i}\hat{S}\left(t_{j-1}\right)\left(t_{j}-t_{j-1}\right)}$$

which is a natural extension of Formula (1) to a scenario involving censoring.

A similar contradiction also occurs for the weighted average of hazards. For form's sake, Formula (4) can be expressed as a natural estimator of Formula (3):

$$\hat{\mathrm{AH}}\left(\tau \right)=\frac{\sum_{j=1}^{i}\hat{S}\left(t_{j-1}\right)\left(t_{j}-t_{j-1}\right)\hat{h}\left(t_{j}\right)+\hat{S}\left(t_{i}\right)\left(\tau -t_{i}\right)\hat{h}\left(\tau \right)}{\sum_{j=1}^{i}\hat{S}\left(t_{j-1}\right)\left(t_{j}-t_{j-1}\right)+\hat{S}\left(t_{i}\right)\left(\tau -t_{i}\right)}$$

This formula for $\hat{\mathrm{AH}}\left(\tau \right)$ implies that $\hat{h}\left(\tau \right)=0$ under the assumption that Formula (4) is correct for $t_{i}<\tau <t_{i+1}$. However, as noted above, $\hat{h}\left(t_{j}\right)$ cannot be defined at a time at which the event is not observed; thus, $\hat{h}\left(\tau \right)$ cannot be defined. This contradiction reveals that the assumption of Formula (4) being correct is false for $t_{i}<\tau <t_{i+1}$.

We note that $\hat{f}\left(\tau \right)$ also cannot be defined for $t_{i}<\tau <t_{i+1}$. This implies that a contradiction similar to $\hat{h}\left(\tau \right)$ above occurs on $\hat{f}\left(\tau \right)$ when the generalized hazard is considered to be an expected number of events within the total survival time.

## 4 Conclusions

The average hazard is the harmonic mean version of the expectation for hazards. This concept is straightforward for researchers to grasp because the average rate can be appropriately derived using the harmonic mean. Furthermore, the average hazard can be estimated using a simple nonparametric method without any underlying assumptions for $\tau =t_{i}$.

Unfortunately, however, for $\tau \neq t_{i}$, the average hazard cannot be estimated appropriately by applying the current method. It would be difficult to develop a nonparametric method to derive an appropriate estimate of the average hazard for $\tau \neq t_{i}$. Although this difficulty could be overcome by applying a parametric model, researchers might want to avoid its application. Further discussion of how $\hat{\mathrm{AH}}\left(\tau \right)$ should be considered for $\tau \neq t_{i}$ will be required.

We advocate for the application of the average hazard in clinical studies and hope that this article encourages its adoption.

## Conflicts of Interest

The author declares no conflicts of interest.

## Data Availability

Data sharing is not applicable to this article as no new data were created or analyzed in this study.
